# Association between Benign Paroxysmal Positional Vertigo and Previous Proton Pump Inhibitor Use: A Nested Case–Control Study Using a National Health Screening Cohort

**DOI:** 10.3390/ijerph191610280

**Published:** 2022-08-18

**Authors:** So Young Kim, Dae Myoung Yoo, Mi Jung Kwon, Ji Hee Kim, Joo-Hee Kim, Joong Seob Lee, Hyo Geun Choi

**Affiliations:** 1 Bundang CHA Medical Center, Department of Otorhinolaryngology—Head and Neck Surgery, CHA University, Seongnam 13488, Korea; 2 Hallym Data Science Laboratory, Hallym University College of Medicine, Anyang 14066, Korea; 3 Department of Pathology, Hallym Sacred Heart Hospital, Hallym University College of Medicine, Anyang 14068, Korea; 4 Department of Neurosurgery, Hallym University College of Medicine, Anyang 14068, Korea; 5 Department of Medicine, Division of Pulmonary, Allergy, and Critical Care Medicine, Hallym Sacred Heart Hospital, Hallym University College of Medicine, Anyang 14068, Korea; 6 Department of Otorhinolaryngology—Head and Neck Surgery, Hallym University College of Medicine, Anyang 14068, Korea

**Keywords:** proton pump inhibitor, benign paroxysmal positional vertigo, risk factors, cohort studies

## Abstract

The present nested case–control study evaluated the impact of previous proton pump inhibitor (PPI) prescription on the risk of benign paroxysmal positional vertigo (BPPV). A ≥40-year-old Korean population was included. A total of 34,441 patients with BPPV was matched with 137,764 comparison participants for demographic and socioeconomic factors. Previous histories of PPI use and PPI prescription dates were compared between the BPPV and comparison groups. The odds ratios (ORs) with 95% confidence intervals (CIs) of PPI use for BPPV were calculated using a logistic regression. The demographic and socioeconomic factors and comorbidities were adjusted in the adjusted model. Both current and past PPI users were associated with higher odds for BPPV than non-PPI users (adjusted OR (aOR) = 3.57, 95% CI = 3.33–3.83, and *p* < 0.001 for current PPI users and aOR = 1.76, 95% CI = 1.64–1.89, and *p* < 0.001 for past PPI users). In addition, longer dates of PPI use were related to higher odds for BPPV (aOR (95% CI) = 1.95 [1.81–2.10] for ≥1 day and <30 days of PPI prescription, <2.88 [2.68–3.10] for ≥30 days and <365 days of PPI prescription, and <3.45 [3.19–3.73] for ≥365 days of PPI prescription). PPI use was linked with an elevated risk of BPPV in the adult population. The odds for BPPV were higher in patients with a longer duration of PPI use.

## 1. Introduction

Proton pump inhibitors (PPIs) are frequently prescribed for a number of diseases, including gastroesophageal reflux disease, gastric or duodenal ulcers, nonsteroidal anti-inflammatory drugs, the eradication of Helicobacter pylori, and erosive esophagitis [1,2]. A PPI is a potent acid suppressor that acts against gastric H/K adenosine triphosphatase by binding to the cysteine residue of the proton pump in gastric parietal cells [1]. The PPI has been acknowledged as a safe drug, and its pleiotropic efficacies have widened the prescription indications of PPIs to respiratory diseases and hypersensitivity [3,4,5]. However, a number of reports have been concerned about the side effects of PPIs [6,7]. It was suspected that the long-term use of PPIs could elevate vulnerability to infections, the hypersecretion of acid due to hypergastrinemia, and malabsorption of micronutrients [7]. In addition, a variety of neurologic diseases was suggested to be increased in PPI users, including Alzheimer dementia, migraines, peripheral neuropathies, and hearing loss [8].

Benign paroxysmal positional vertigo (BPPV) is a peripheral vestibular disorder with vertigo triggered by specific head positional changes [9,10,11]. BPPV is a common peripheral vestibular disorder whose prevalence is predicted to be approximately 2.4% [12]. The pathophysiology of BPPV has been attributed to otoconia dislocated from otolith organs, which perturb the endolymphatic flow in the semicircular canals according to head position [13,14]. Therefore, inner ear dysfunctions, for instance, the disturbance of endolymphatic homeostasis or degenerative insults of otoconia, can induce BPPV. A few etiologic factors have been mentioned for the occurrence of BPPV. Deficits in vitamin D and osteoporosis, which can influence degenerative otoconial changes, have been suggested as risk factors for BPPV [15,16]. Several chronic diseases, such as hypertension, diabetes mellitus, and hyperlipidemia, were also reported to increase the risk of BPPV [16]. PPI use has been suggested to be related to inner ear disorders of hearing loss [17]. In addition, the influence of PPI use on inner ear homeostasis has been postulated [18]. However, to our knowledge, the association of PPI use with the occurrence of BPPV has not yet been explored.

The current study supposed that PPI use could impact the occurrence of BPPV. To examine this assumption, the national cohort population was analyzed for the occurrence of BPPV according to the history of PPI prescription. Because prior reports mentioned the adverse impacts of the long-term use of PPIs, the duration of PPI use was considered for the occurrence of BPPV in this study. Moreover, numerous variables of demographic and comorbid conditions were considered and further analyzed to elucidate the differentiated impact of PPI use on the occurrence of BPPV.

## 2. Materials and Methods

### 2.1. Data Source and Ethical Consideration

The Korean National Health Insurance Service-Health Screening Cohort (NHIS-HEALS) data were analyzed in the current study. The NHIS-HEALS included participants who conducted health screenings which were supported by the NHIS [19]. The NHIS sampled the representative national cohort by randomly selecting 10% of the health screening population [19]. The NHIS-HEALS data encompassed both the health screening data and the health claim data [19]. The Institutional Review Board (IRB) of Hallym University (IRB no.: 2019-10-023) approved the current research. The IRB exempted written informed consent. This study followed the Strengthening the Reporting of Observational Studies in Epidemiology (STROBE) statement for reporting.

### 2.2. Participant Selection

**Figure 1:**
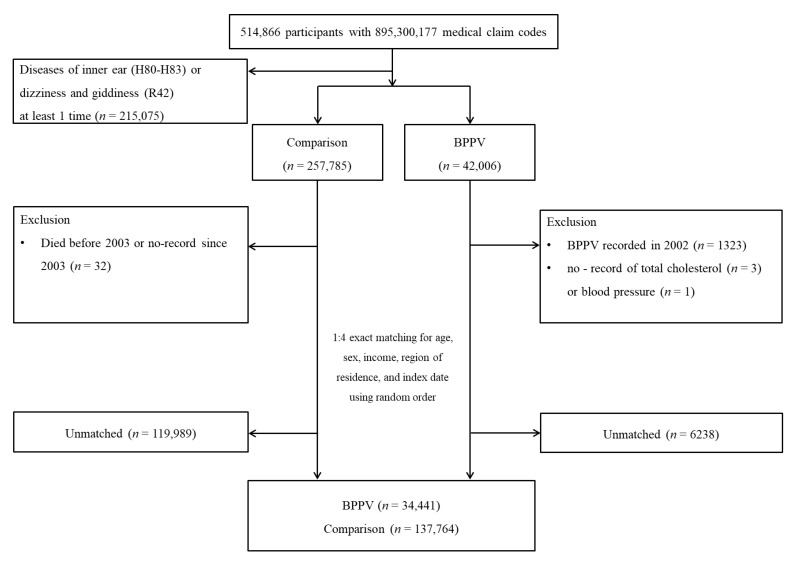
A schematic illustration of the participant selection process used in the present study. Of a total of 514,866 participants, 34,441 BPPV participants were matched with 137,764 comparison participants for age, sex, income, and region of residence.

Among a total population of 514,866 participants, 42,006 were enrolled into the BPPV group (*n* = 42,006). Participants who were defined as having BPPV in 2002 among the BPPV group were excluded (washout, *n* = 1323). Participants who were without a record of total cholesterol (*n* = 3) or blood pressure (*n* = 1) were excluded. Participants were also excluded if they were diagnosed with H80 to H83 (diseases of the inner ear) or R42 (dizziness and giddiness) at least 1 time using the ICD-10 codes (*n* = 215,075). Among the comparison group (*n* = 257,785), participants were removed if they had died before 2003 or had no records since 2003 (*n* = 32). The BPPV participants were matched with comparison participants for age, sex, income, and region of residence. The control participants were designated in random number order. The index date of each BPPV participant was set as the time of treatment of BPPV. The index date of the comparison participants was defined as the index date of their matched BPPV participants. Finally, 34,441 BPPV participants were 1:4 matched with 137,764 comparison participants (Figure 1).

#### 2.2.1. Proton Pump Inhibitor (Exposure)

Proton pump inhibitor (PPI) users were classified based on the history of PPI prescription within a year. All types of PPIs were included based on the history of prescription. The history of PPI use was examined: (1) PPI usage history and (2) PPI prescription dates.

In the first category, current PPI users were defined as participants who were prescribed PPIs within 30 days. Past PPI users were defined as participants who were prescribed PPIs within 31 days to 365 days. Others were defined as PPI nonusers.

In the second category, the participants were classified into four groups as PPI nonusers, 1 day ≤ PPI prescription dates < 30 days, 30 days ≤ PPI prescription dates < 1 year (365 days), and PPI prescription dates ≥ 1 year (365 days).

#### 2.2.2. Benign Paroxysmal Positional Vertigo (Outcome)

History of BPPV was classified for participants with ≥2 times of treatment histories [20].

#### 2.2.3. Covariates

Age was classified into 10 groups (40–44, 45–49, 50–54, 55–59, 60–64, 65–69, 70–74, 75–79, 80–84, and 85 or more years old) [21]. The level of income was categorized into groups 1 (lowest), 2, 3, 4, and 5 (highest) [22]. The regions of residence were classified into urban and rural groups [22]. Tobacco smoking was categorized into nonsmoker, past smoker, and current smoker [23]. Alcohol consumption was categorized into less than 1 time a week and 1 or more times a week of alcohol consumption [23]. Obesity using body mass index (BMI, kg/m^2^) was categorized into underweight, normal, overweight, obese I, and obese II [23]. Total cholesterol (mg/dL), systolic blood pressure (SBP, mmHg), diastolic blood pressure (DBP, mmHg), and fasting blood glucose (mg/dL) were measured and analyzed as continuous variables. The Charlson comorbidity index (CCI) was categorized into groups with 0, 1, and 2 or more scores [24].

Osteoporosis was defined based on the ICD-10 codes, ≥2 times of treatment histories, and dual energy X-ray absorptiometry (DEXA) or DEXA CT scan examination [25].

The diagnosis of gastroesophageal reflux disease (GERD, K21 for ICD-10 codes ≥ 2 times with PPI prescription dates ≥ 2 weeks) was counted. H2 blocker dates were summed.

### 2.3. Statistical Analyses

We conducted propensity score (PS) overlap weighting to reflect the covariate balance and effective sample size. The PS was calculated with a multivariable logistic regression with all covariates. To calculate the overlap weighting, the PS was applied, in which each BPPV participant was weighted with the probability of the PS and each control participant was weighted with the probability of 1-PS. Overlap weighting was calculated between 0 and 1 and achieved exact balance and optimized precision [26,27,28]. The standardized difference (sd) with overlap weighting was used to compare the difference in general characteristics between the BPPV and control groups.

A logistic regression was used to analyze the odds ratios (ORs) with 95% confidence intervals (CIs) of PPI users and of PPI prescription dates for BPPV. In these analyses, crude and adjusted models were used. These models were adjusted for age, sex, income, region of residence, total cholesterol, SBP, DBP, fasting blood glucose, CCI score, osteoporosis diagnosis, prescription dates of H2 blockers within 1 year, and the number of GERD treatments within 1 year in the adjusted model. A PS overlap-weighted logistic regression was applied in these models.

Subgroup analyses according to age, sex, income, region of residence, obesity, smoking status, alcohol consumption, total cholesterol, blood pressure, and fasting blood glucose were performed.

SAS version 9.4 (SAS Institute Inc., Cary, NC, USA) was utilized. Two-tailed analyses were conducted. The *p*-value < 0.05 was defined as the statistical significance.

## 3. Results

A total of 67.3% (23,169/34,441) of the BPPV group and 45.8% (63,031/137,764) of the comparison group was current PPI users (sd = 0.47, Table 1). The PPI prescription dates were longer in the BPPV group than in the comparison group (mean = 234.8 (standard deviation (SD)) = 243.6 days vs. 167.7 (SD = 206.1) days, sd = 0.30). The H2 blocker prescription dates, number of GERD treatments, CCI scores, and histories of osteoporosis were also higher in the BPPV group than in the comparison group. After the overlap weighting adjustment, 66.4% and 48.7% of the BPPV and comparison groups, respectively, were current PPI users (sd = 0.39). The PPI prescription dates were still longer in the BPPV group than in the comparison group after the overlap weighting adjustment (226.4 (SD = 209.1) vs. 185.5 (SD = 96.1), sd = 0.25).

Current PPI users demonstrated 3.57-fold higher odds for BPPV than non-PPI users in the adjusted model with overlap weighting (95% CI = 3.33–3.83, *p* < 0.001, Table 2). Past PPI users also indicated higher odds for BPPV (adjusted OR (aOR) = 1.76, 95% CI = 1.64–1.89, *p* < 0.001). According to the dates of PPI prescription, longer dates of PPI prescription were associated with higher odds for BPPV (aOR (95% CI) = 1.95 [1.81–2.10] < 2.88 [2.68–3.10] < 3.45 [3.19–3.73] for ≥1 day and <30 days, ≥30 days and <365 days, and ≥365 days of PPI prescription).

**Figure 2:**
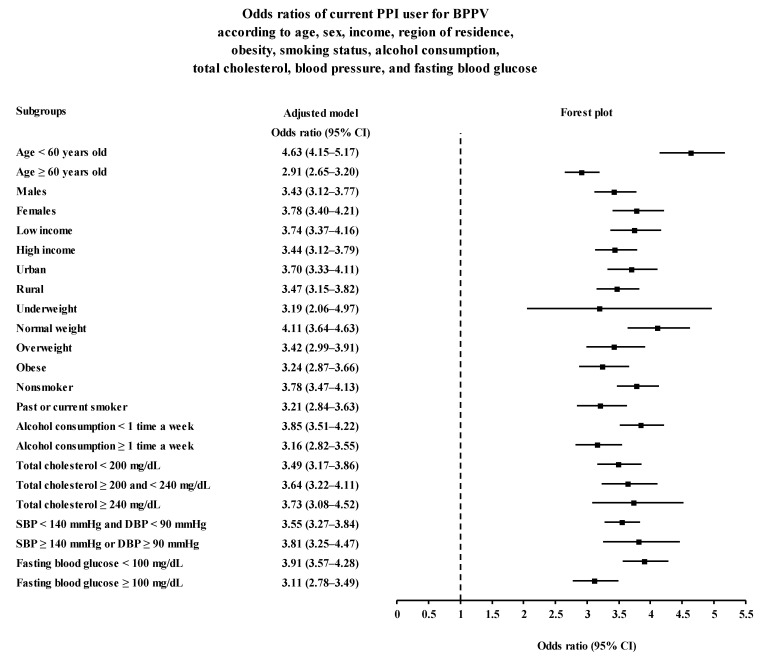
Subgroup analyses of current PPI users for BPPV according to age, sex, income, region of residence, obesity, smoking status, alcohol consumption, total cholesterol, blood pressure, and fasting blood glucose visualized with forest plot. Abbreviations: BPPV, benign paroxysmal positional vertigo; DBP, diastolic blood pressure; PPI, proton pump inhibitor; SBP, systolic blood pressure.

**Figure 3:**
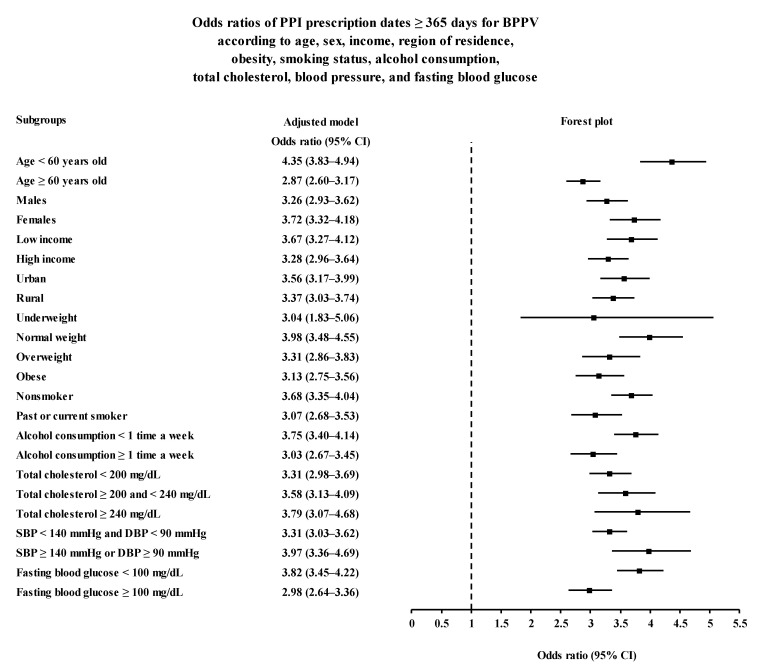
Subgroup analyses of PPI prescription dates ≥ 365 for BPPV according to age, sex, income, region of residence, obesity, smoking status, alcohol consumption, total cholesterol, blood pressure, and fasting blood glucose visualized with forest plot. Abbreviations: BPPV, benign paroxysmal positional vertigo; DBP, diastolic blood pressure; PPI, proton pump inhibitor; SBP, systolic blood pressure.

The positive association between PPI use and BPPV was consistent in all subgroup analyses according to age, sex, income, region of residence, BMI group, smoking, alcohol consumption, cholesterol level, SBP, and fasting blood glucose level (Figure 2 and Figure 3 and Supplementary Tables S1 and S2).

## 4. Discussion

PPI use was associated with a higher risk of BPPV in the adult population. In particular, a longer duration of PPI use was related to greater odds for BPPV. The relationship of PPI use with BPPV was maintained in all subgroups in accordance with the demographic and comorbid conditions. This study improved previous findings on the potential adverse effects of PPIs on inner ear diseases.

Although there was no prior research on the impact of PPI use on BPPV, a few previous studies addressed the adverse impacts of PPI use on inner ear diseases with controversial results [17,29]. The long-term use of PPIs was suggested to accelerate the progression of hearing loss [29]. In addition, other case-control studies reported a higher risk of hearing loss in PPI users [30,31]. Compared to non-PPI users, PPI users demonstrated a 1.50 times higher risk of hearing loss or tinnitus [30]. On the other hand, a prospective study demonstrated no relation in PPI use with the risk of hearing loss [17]. Instead, they reported the association of GERD symptoms with a greater risk of hearing loss (multivariable-adjusted relative risks = 1.33, 95% CI = 1.19–1.49, *p* < 0.001) [17]. However, that study included only a female population with a limited range of ages (41–58 years old) [17]. Only one retrospective study evaluated the relationship of PPI use with a peripheral vestibular disorder [32]. That study reported less frequent Meniere attacks in PPI users than in non-PPI users [32]. However, that study had as few as 42 participants of the study population with Meniere’s disease, which lacked control participants.

The regulatory roles of PPIs on endolymphatic homeostasis could have mediated the relationship of PPI use with the occurrence of BPPV in this study. The expression of gastric-type proton pumps (H,K-ATPase) was described in the vestibular organs of the utricle, saccule, and ampulla [33,34]. This proton pump was presumed to be crucial to regulate potassium circulation in the endolymphatic space [33]. Thus, PPI use could modulate the endolymphatic homeostasis of semicircular canals and otolith organs, thereby increasing the risk of BPPV.

In addition, the increased risk of otoconial degeneration due to osteoporotic changes could be linked with the risk of BPPV in PPI users. A higher risk of osteoporosis was reported in long-term PPI users [35,36]. The long-term PPI prescription could suppress the adequate absorption of calcium and vitamins, which would result in a reduced bone mineral density [35]. Osteoporosis is known to be a risk factor for BPPV [37,38]. It was reported that patients with osteoporosis were exposed to a 1.28 times greater rate of occurrence of BPPV (95% CI = 1.16–1.42) [38]. The degenerative changes in otoconia in an osteoporotic animal model supported the impact of osteoporosis on the development of BPPV [39].

Other cardiovascular and metabolic changes according to chronic PPI use could also explain the higher risk of BPPV in PPI users [40]. The long-term use of PPIs was related to a higher risk of cardiovascular diseases, including stroke, coronary heart disease, and heart failure (hazard ratio = 2.02, 95% CI = 1.50–2.72) [40]. Although there were some conflicts, the occurrence or recurrence of BPPV was reported to be associated with cardiovascular risk factors, such as hyperlipidemia and hypertension [41,42]. Moreover, carotid stenosis and inflammatory markers of interleukin-1β soluble vascular adhesion protein-1 were supposed to be related to the pathogenesis of BPPV [43]. Thus, the long-term use of PPIs could increase the risk of BPPV by inducing cardiovascular compromises, which could result in inflammatory and ischemic insults in vestibular organs.

This study newly demonstrated the possible harmful effect of PPI use on the occurrence of BPPV. The large study population and matched control participants enhanced the reliability of the current results. Histories of other chronic diseases, including GERD, were considered to attenuate the potential confounding effects. Histories of PPI use were obtained from health claim data, and the durations of prescriptions were analyzed to examine the impact of the long-term use of PPIs on BPPV. However, the types of PPIs were heterogeneous in the present study. The drug metabolism of PPIs was addressed to be different according to specific types of PPIs [2]. Thus, the impacts of PPIs on the occurrence of BPPV could be different according to the types of PPIs. In addition, the types of BPPV could not be differentiated in the current study. The findings of the vestibular function test were not available in the present cohort. Although several conditions that could be related with dizziness were excluded in this study, the potential for a mixed condition with other causes, such as middle ear cholesteatoma, head injuries, and fractures of the temporal bone, was not totally removed. Finally, this study had a nested case–control design, and we could not conclude the causality between PPI use and BPPV. Although we randomly selected the comparison participants, selection bias could not be totally excluded. Future studies with prospective study designs could solve the current questions.

## 5. Conclusions

The prior use of PPIs was related to a higher risk of BPPV in the adult population. The longer use of PPIs was associated with a greater risk of BPPV. The potential adverse impacts of PPIs on BPPV need to be considered when prescribing PPIs in patients with BPPV.

## Figures and Tables

**Table 1:** General characteristics of participants before and after propensity score overlap weighting adjustment.

Characteristics	Before Overlap Weighting Adjustment		Standardized Difference	After Overlap Weighting Adjustment		Standardized Difference
	BPPV	Comparison		BPPV	Comparison	
Number	34,441	137,764		26,737	26,737	
Age (years, mean, SD)	61.9 (9.1)	61.6 (9.0)	0.03	61.9 (8.0)	61.6 (4.0)	0.04
Age (years, *n*, %)			0.00			0.03
40–44	460 (1.3)	1840 (1.3)		359 (1.3)	366 (1.4)	
45–49	2299 (6.7)	9196 (6.7)		1799 (6.7)	1804 (6.8)	
50–54	4871 (14.1)	19,484 (14.1)		3812 (14.3)	3780 (14.1)	
55–59	6928 (20.1)	27,712 (20.1)		5417 (20.3)	5301 (19.8)	
60–64	7280 (21.1)	29,120 (21.1)		5649 (21.1)	5664 (21.2)	
65–69	5704 (16.6)	22,816 (16.6)		4368 (16.3)	4535 (17.0)	
70–74	3551 (10.3)	14,204 (10.3)		2721 (10.2)	2787 (10.4)	
75–79	2152 (6.3)	8608 (6.3)		1670 (6.2)	1635 (6.1)	
80–84	964 (2.8)	3856 (2.8)		758 (2.8)	698 (2.6)	
85+	232 (0.7)	928 (0.7)		186 (0.7)	167 (0.6)	
Sex (*n*, %)			0.00			0.00
Males	14,694 (42.7)	58,776 (42.7)		11,399 (42.6)	11,399 (42.6)	
Females	19,747 (57.3)	78,988 (57.3)		15,338 (57.4)	15,338 (57.4)	
Income (*n*, %)			0.00			0.01
1 (lowest)	5658 (16.4)	22,632 (16.4)		4356 (16.3)	4386 (16.4)	
2	4492 (13.0)	17,968 (13.0)		3490 (13.1)	3454 (12.9)	
3	5408 (15.7)	21,632 (15.7)		4201 (15.7)	4172 (15.6)	
4	7454 (21.6)	29,816 (21.6)		5785 (21.6)	5829 (21.8)	
5 (highest)	11,429 (33.2)	45,716 (33.2)		8905 (33.3)	8896 (33.3)	
Region of residence (*n*, %)			0.00			0.00
Urban	15,318 (44.5)	61,272 (44.5)		11,914 (44.6)	11,914 (44.6)	
Rural	19,123 (55.5)	76,492 (55.5)		14,823 (55.4)	14,823 (55.4)	
Total cholesterol level (mg/dL, mean, SD)	199.6 (38.2)	200.0 (38.9)	0.01	199.7 (33.6)	199.7 (17.1)	0.00
SBP (mmHg, mean, SD)	126.3 (16.4)	126.7 (17.2)	0.02	126.3 (14.5)	126.3 (7.5)	0.00
DBP (mmHg, mean, SD)	77.8 (10.4)	78.0 (10.9)	0.01	77.9 (9.2)	77.9 (4.8)	0.00
Fasting blood glucose level (mg/dL, mean, SD)	99.9 (26.7)	101.4 (30.1)	0.05	100.2 (24.2)	100.2 (11.8)	0.00
Obesity (*n*, %) ^‡^			0.08			0.00
Underweight	711 (2.1)	3689 (2.7)		577 (2.2)	577 (2.2)	
Normal	11,613 (33.7)	50,402 (36.6)		9172 (34.3)	9172 (34.3)	
Overweight	9760 (28.3)	36,894 (26.8)		7500 (28.1)	7500 (28.1)	
Obese I	11,288 (32.8)	42,333 (30.7)		8653 (32.4)	8653 (32.4)	
Obese II	1069 (3.1)	4446 (3.2)		836 (3.1)	836 (3.1)	
Smoking status (*n*, %)			0.13			0.00
Nonsmoker	26,432 (76.8)	102,093 (74.1)		20,395 (76.3)	20,395 (76.3)	
Past smoker	4620 (13.4)	16,483 (12.0)		3523 (13.2)	3523 (13.2)	
Current smoker	3389 (9.8)	19,188 (13.9)		2819 (10.5)	2819 (10.5)	
Alcohol consumption (*n*, %)			0.09			0.00
<1 time a week	24,574 (71.4)	92,741 (67.3)		18,851 (70.5)	18,851 (70.5)	
≥1 time a week	9867 (28.7)	45,023 (32.7)		7886 (29.5)	7886 (29.5)	
CCI score (score, mean, SD)	1.1 (1.7)	0.9 (1.7)	0.09	1.0 (1.4)	1.0 (0.8)	0.00
CCI score (*n*, %)			0.20			0.15
0 score	18,851 (54.7)	88,690 (64.4)		14,878 (55.6)	16,525 (61.8)	
1 score	6798 (19.7)	19,354 (14.1)		5257 (19.7)	3826 (14.3)	
≥2 scores	8792 (25.5)	29,720 (21.6)		6603 (24.7)	6386 (23.9)	
Osteoporosis (*n*, %)	11,131 (32.3)	32,456 (23.6)	0.20	8064 (30.2)	8064 (30.2)	0.00
H2 blocker prescription dates (days, mean, SD)	29.1 (62.4)	16.5 (49.9)	0.22	25.2 (48.4)	25.2 (29.8)	0.00
No. of GERD treatments (no., mean, SD)	0.7 (2.3)	0.3 (1.4)	0.22	0.6 (1.5)	0.6 (1.0)	0.00
No. of GERD treatments (*n*, %)			0.32			0.17
0 time	26,696 (77.5)	122,936 (89.2)		21,256 (79.5)	22,857 (85.5)	
1 time	2865 (8.3)	6013 (4.4)		2198 (8.2)	1275 (4.8)	
≥2 times	4880 (14.2)	8815 (6.4)		3283 (12.3)	2605 (9.7)	
PPI prescription history (*n*, %)			0.47			0.39
PPI nonuser	1574 (4.6)	17,662 (12.8)		1283 (4.8)	3010 (11.3)	
Past PPI user	9698 (28.2)	57,071 (41.4)		7700 (28.8)	10,713 (40.1)	
Current PPI user	23,169 (67.3)	63,031 (45.8)		17,754 (66.4)	13,015 (48.7)	
PPI prescription dates (days, mean, SD)	234.8 (243.6)	167.7 (206.1)	0.30	226.4 (209.1)	185.5 (96.1)	0.25
PPI prescription dates (*n*, %)			0.39			0.29
PPI nonuser	1574 (4.6)	17,662 (12.8)		1283 (4.8)	3010 (11.3)	
≥1 day and <30 days PPI user	7416 (21.5)	40,139 (29.1)		5959 (22.3)	7264 (27.2)	
≥30 days and < 365 days PPI user	15,327 (44.5)	52,642 (38.2)		11,932 (44.6)	10,483 (39.2)	
≥365 days PPI user	10,124 (29.4)	27,321 (19.8)		7563 (28.3)	5980 (22.4)	

Abbreviations: BPPV, benign paroxysmal positional vertigo; CCI, Charlson comorbidity index; DBP, diastolic blood pressure; GERD, gastroesophageal reflux disease; PPI, proton pump inhibitor; SBP, systolic blood pressure; SD, standard deviation. ^‡^ Obesity (BMI, body mass index, kg/m^2^) was categorized as <18.5 (underweight), ≥18.5 to <23 (normal), ≥23 to <25 (overweight), ≥25 to <30 (obese I), and ≥30 (obese II).

**Table 2:** Odds ratios (95% confidence interval) of PPI prescription history and PPI prescription dates for BPPV.

Characteristics	BPPV	Comparison	Odds Ratios (95% Confidence Intervals)			
	(Exposure/Total, %)	(Exposure/Total, %)	Crude	*p*-Value	Adjusted Model with OW ^†^	*p*-Value
User of PPI						
Past PPI user	9698/66,769 (14.5)	57,071/66,769 (85.5)	1.91 (1.80–2.02)	<0.001 *	1.76 (1.64–1.89)	<0.001 *
Current PPI user	23,169/86,200 (26.9)	63,031/86,200 (73.1)	4.12 (3.91–4.35)	<0.001 *	3.57 (3.33–3.83)	<0.001 *
PPI dates						
≥1 day and <30 days	7416/47,555 (15.6)	40,139/47,555 (84.4)	2.07 (1.96–2.19)	<0.001 *	1.95 (1.81–2.10)	<0.001 *
≥30 days and < 365 days	15,327/67,969 (22.6)	52,642/67,969 (77.5)	3.27 (3.09–3.45)	<0.001 *	2.88 (2.68–3.10)	<0.001 *
≥365 days	10,124/37,445 (27.0)	27,321/37,445 (73.0)	4.16 (3.93–4.40)	<0.001 *	3.45 (3.19–3.73)	<0.001 *

Abbreviations: BPPV, benign paroxysmal positional vertigo; CCI, Charlson comorbidity index; DBP, diastolic blood pressure; GERD, gastroesophageal reflux disease; OW, overlap weighting; PPI, proton pump inhibitor; SBP, systolic blood pressure. * Logistic regression model, significance at *p* < 0.05. ^†^ Adjusted for age, sex, income, region of residence, obesity, smoking status, alcohol consumption, total cholesterol, SBP, DBP, fasting blood glucose, CCI score, osteoporosis diagnosis, prescription dates of H2 blockers, and the number of GERD treatments.

## Data Availability

Restrictions apply to the availability of these data. Data were obtained from the Korean National Health Insurance Sharing Service (NHISS) and are available at https://nhiss.nhis.or.kr (accessed on 10 June 2019) with the permission of the NHISS.

## Supplementary Materials

The following supporting information can be downloaded at: https://www.mdpi.com/article/10.3390/ijerph191610280/s1, Table S1. Subgroup analyses regarding odds ratio (95% confidence intervals) of PPI prescription history for BPPV according to age, sex, income, region of residence, obesity, smoking state, alcohol consumption, total cholesterol, blood pressure, and fasting blood glucose. Table S2. Subgroup analyses regarding odds ratio (95% confidence intervals) of PPI prescription dates for BPPV according to age, sex, income, region of residence, obesity, smoking state, alcohol consumption, total cholesterol, blood pressure, and fasting blood glucose.

## References

[1] Chubineh S., Birk J. (2012). Proton Pump Inhibitors. South. Med. J..

[2] Shi S., Klotz U. (2008). Proton pump inhibitors: An update of their clinical use and pharmacokinetics. Eur. J. Clin. Pharmacol..

[3] Hassall E. (2012). Over-Prescription of Acid-Suppressing Medications in Infants: How It Came About, Why It’s Wrong, and What to Do About It. J. Pediatr..

[4] Ummarino D., Miele E., Masi P., Tramontano A., Staiano A., Vandenplas Y. (2012). Impact of antisecretory treatment on respiratory symptoms of gastroesophageal reflux disease in children. Dis. Esophagus.

[5] Tofil N.M., Benner K.W., Fuller M.P., Winkler M.K. (2008). Histamine 2 receptor antagonists vs intravenous proton pump inhibitors in a pediatric intensive care unit: A comparison of gastric pH. J. Crit. Care.

[6] Haastrup P.F., Thompson W., Søndergaard J., Jarbøl D. (2018). Side Effects of Long-Term Proton Pump Inhibitor Use: A Review. Basic Clin. Pharmacol. Toxicol..

[7] Ghosh G., Schnoll-Sussman F., Mathews S., Katz P.O. (2020). Reported proton pump inhibitor side effects: What are physician and patient perspectives and behaviour patterns?. Aliment. Pharmacol. Ther..

[8] Makunts T., Alpatty S., Lee K.C., Atayee R.S., Abagyan R. (2019). Proton-pump inhibitor use is associated with a broad spectrum of neurological adverse events including impaired hearing, vision, and memory. Sci. Rep..

[9] Kim J.-S., Zee D.S. (2014). Benign Paroxysmal Positional Vertigo. N. Engl. J. Med..

[10] Bhattacharyya N., Gubbels S.P., Schwartz S.R., Edlow J.A., El-Kashlan H., Fife T., Holmberg J.M., Mahoney K., Hollingsworth D.B., Roberts R. (2017). Clinical Practice Guideline: Benign Paroxysmal Positional Vertigo (Update). Otolaryngol. Neck Surg..

[11] Shim D.B. (2020). Treatment of Benign Paroxysmal Positional Vertigo: An Approach Considering Patients’ Convenience. Clin. Exp. Otorhinolaryngol..

[12] Von Brevern M., Radtke A., Lezius F., Feldmann M., Ziese T., Lempert T., Neuhauser H. (2007). Epidemiology of benign paroxysmal positional vertigo: A population based study. J. Neurol. Neurosurg. Psychiatry.

[13] Parnes L.S., Agrawal S.K., Atlas J. (2003). Diagnosis and management of benign paroxysmal positional vertigo (BPPV). Can. Med Assoc. J..

[14] Lee H.J., Jeon E.-J., Lee D.-H., Seo J.-H. (2020). Therapeutic Efficacy of the Modified Epley Maneuver with a Pillow Under the Shoulders. Clin. Exp. Otorhinolaryngol..

[15] Kim H.-J., Park J., Kim J.-S. (2021). Update on benign paroxysmal positional vertigo. J. Neurol..

[16] Chen J., Zhang S., Cui K., Liu C. (2021). Risk factors for benign paroxysmal positional vertigo recurrence: A systematic review and meta-analysis. J. Neurol..

[17] Lin B.M., Curhan S.G., Wang M., Jacobson B.C., Eavey R., Stankovic K.M., Curhan G.C. (2017). Prospective Study of Gastroesophageal Reflux, Use of Proton Pump Inhibitors and H2-Receptor Antagonists, and Risk of Hearing Loss. Ear Hear..

[18] Pirodda A., Brandolini C., Raimondi M.C., Modugno G.C. (2009). The possible role of proton pump inhibitors of the homeostasis of the inner ear. Med. Hypotheses.

[19] Seong S.C., Kim Y.-Y., Park S.K., Khang Y.-H., Kim H.C., Park J.H., Kang H.-J., Do C.-H., Song J.-S., Lee E.-J. (2017). Cohort profile: The National Health Insurance Service-National Health Screening Cohort (NHIS-HEALS) in Korea. BMJ Open.

[20] Kim S.K., Hong S.M., Park I.-S., Choi H.G. (2019). Association Between Migraine and Benign Paroxysmal Positional Vertigo Among Adults in South Korea. JAMA Otolaryngol. Neck Surg..

[21] Kim S.Y., Min C., Yoo D.M., Chang J., Lee H.-J., Park B., Choi H.G. (2021). Hearing Impairment Increases Economic Inequality. Clin. Exp. Otorhinolaryngol..

[22] Kim S.Y., Min C., Oh D.J., Choi H.G. (2020). Bidirectional Association Between GERD and Asthma: Two Longitudinal Follow-Up Studies Using a National Sample Cohort. J. Allergy Clin. Immunol. Pr..

[23] Kim S.Y., Oh D.J., Park B., Choi H.G. (2020). Bell’s palsy and obesity, alcohol consumption and smoking: A nested case-control study using a national health screening cohort. Sci. Rep..

[24] Quan H., Li B., Couris C.M., Fushimi K., Graham P., Hider P., Januel J.-M., Sundararajan V. (2011). Updating and Validating the Charlson Comorbidity Index and Score for Risk Adjustment in Hospital Discharge Abstracts Using Data From 6 Countries. Am. J. Epidemiol..

[25] Kim S.Y., Kong I.G., Lim H., Choi H.G. (2018). Increased Risk of Sudden Sensory Neural Hearing Loss in Osteoporosis: A Longitudinal Follow-Up Study. J. Clin. Endocrinol. Metab..

[26] Li F., E Thomas L. (2019). Addressing Extreme Propensity Scores via the Overlap Weights. Am. J. Epidemiology.

[27] Thomas L.E., Li F., Pencina M.J. (2020). Overlap Weighting. JAMA.

[28] Zhu Y., Schonbach M., Coffman D.L., Williams J.S. (2015). Variable Selection for Propensity Score Estimation via Balancing Covariates. Epidemiology.

[29] Wiciński M., Malinowski B., Puk O., Górski K., Adamkiewicz D., Chojnacki G., Walczak M., Wódkiewicz E., Szambelan M., Adamska P. (2019). Possible Effects of Proton Pump Inhibitors on Hearing Loss Development. BioMed Res. Int..

[30] Yee J., Han H.W., Gwak H.S. (2022). Proton pump inhibitor use and hearing loss in patients with type 2 diabetes: Evidence from a hospital-based case-control study and a population-based cohort study. Br. J. Clin. Pharmacol..

[31] Kim S., Lee C., Min C., Yoo D., Choi H. (2021). Association between Proton Pump Inhibitors and Hearing Impairment: A Nested Case-Control Study. Curr. Issues Mol. Biol..

[32] Pirodda A., Modugno G.C., Manzari L., Raimondi M.C., Brandolini C., Ferri G.G., Borghi C. (2010). Meniere’s disease and the use of proton pump inhibitors. Swiss Med. Wkly..

[33] Takumida M., Takumida H., Anniko M. (2016). Gastric-type H+,K+-ATPase in mouse vestibular end organs. Acta Oto-Laryngol..

[34] Lecain E., Robert J.-C., Thomas A., Huy P.T.B. (2000). Gastric proton pump is expressed in the inner ear and choroid plexus of the rat. Hear. Res..

[35] Maléth J., Hegyi P. (2013). Long-term proton pump inhibitor therapy and osteoporosis. Is there a real danger?. Orvosi Hetil..

[36] Lin S.-M., Yang S.-H., Liang C.-C., Huang H.-K. (2018). Proton pump inhibitor use and the risk of osteoporosis and fracture in stroke patients: A population-based cohort study. Osteoporos. Int..

[37] Guo T., Xing Y., Zhu H., Yang L., Xiao Y., Xu J. (2021). Relationship between osteoporosis and benign paroxysmal positional vertigo based on evidence-based medicine and bioinformatics. Arch. Osteoporos..

[38] Kim S.Y., Kim H.-J., Min C., Choi H.G. (2020). Association between benign paroxysmal positional vertigo and osteoporosis: Two nested case-control studies. Osteoporos. Int..

[39] Vibert D., Sans A., Kompis M., Travo C., Mühlbauer R.C., Tschudi I., Boukhaddaoui H., Häusler R. (2008). Ultrastructural Changes in Otoconia of Osteoporotic Rats. Audiol. Neurotol..

[40] Bell E.J., Bielinski S.J., Sauver J.L.S., Chen L.Y., Rooney M.R., Larson N.B., Takahashi P.Y., Folsom A.R. (2021). Association of Proton Pump Inhibitors with Higher Risk of Cardiovascular Disease and Heart Failure. Mayo Clin. Proc..

[41] Sfakianaki I., Binos P., Karkos P., Dimas G.G., Psillas G. (2021). Risk Factors for Recurrence of Benign Paroxysmal Positional Vertigo. A Clinical Review. J. Clin. Med..

[42] Singh J.M., Corser W.D., Monsell E.M. (2020). Cardiovascular Risk Factors and Benign Paroxysmal Positional Vertigo in Community Otolaryngology–Head and Neck Surgery. Otolaryngol. Neck Surg..

[43] Chen X., Feng H., Liu H., Xu X., Wang J., Jin Z. (2020). Carotid imaging changes and serum IL-1β, sICAM-1, and sVAP-1 levels in benign paroxysmal positional vertigo. Sci. Rep..

