# Supplementary material for: Association between Benign Paroxysmal Positional Vertigo and Previous Proton Pump Inhibitor Use: A Nested Case–Control Study Using a National Health Screening Cohort
Source: Int J Environ Res Public Health. 2022 Aug 18;19(16):10280. doi: 10.3390/ijerph191610280 (PMC9408034; doi:10.3390/ijerph191610280)
Supplement: Supplementary file 1 [file ijerph-19-10280-s001.zip › ijerph-1849466-supplementary.pdf]

Table S1. Subgroup analyses regarding odds ratio (95% confidence intervals) of PPI prescription history for BPPV according to age, sex, income, region of residence, obesity, smoking state, alcohol consumption, total cholesterol, blood pressure, and fasting blood glucose.

PPI Prescription History	BPPV	Comparison	Odds ratios (95% Confidence Intervals)			
	(Exposure/Total, %)	(Exposure/Total, %)	Crude	*p*-Value	Adjusted Model with OW †	*p*-Value
Age < 60 years old (n = 72,790)						
Past PPI user	4645/32,295 (14.4)	27,650/32,295 (85.6)	2.20 (2.02–2.40)	<0.001*	2.04 (1.83–2.28)	<0.001*
Current PPI user	9292/31,737 (29.3)	22,445/31,737 (70.7)	5.43 (4.98–5.91)	<0.001*	4.63 (4.15–5.17)	<0.001*
Age ≥ 60 years old (n = 99,415)						
Past PPI user	5053/34,474 (14.7)	29,421/34,474 (85.3)	1.72 (1.60–1.85)	<0.001*	1.57 (1.43–1.73)	<0.001*
Current PPI user	13,877/54,463 (25.5)	40,586/54,463 (74.5)	3.42 (3.19–3.66)	<0.001*	2.91 (2.65–3.20)	<0.001*
Males (n = 73,470)						
Past PPI user	4397/28,621 (15.4)	24,224/28,621 (84.6)	1.86 (1.73–2.00)	<0.001*	1.75 (1.59–1.93)	<0.001*
Current PPI user	9368/34,399 (27.2)	25,031/34,399 (72.8)	3.84 (3.57–4.12)	<0.001*	3.43 (3.12–3.77)	<0.001*
Females (n = 98,735)						
Past PPI user	5301/38,148 (13.9)	32,847/38,148 (86.1)	2.04 (1.87–2.22)	<0.001*	1.81 (1.62–2.02)	<0.001*
Current PPI user	13,801/51,801 (26.6)	38,000/51,801 (73.4)	4.58 (4.22–4.98)	<0.001*	3.78 (3.40–4.21)	<0.001*
Low income (n = 77,790)						
Past PPI user	4283/29,501 (14.5)	25,218/29,501 (85.5)	2.03 (1.87–2.21)	<0.001*	1.86 (1.67–2.07)	<0.001*
Current PPI user	10,576/39,237 (27.0)	28,661/39,237 (73.1)	4.41 (4.07–4.78)	<0.001*	3.74 (3.37–4.16)	<0.001*
High income (n = 94,415)						
Past PPI user	5415/37,268 (14.5)	31,853/37,268 (85.5)	1.81 (1.68–1.95)	<0.001*	1.68 (1.53–1.85)	<0.001*
Current PPI user	12,593/46,963 (26.8)	34,370/46,963 (73.2)	3.90 (3.63–4.19)	<0.001*	3.44 (3.12–3.79)	<0.001*
Urban (n = 76,590)						

Past PPI user	4374/29,919 (14.6)	25,545/29,919 (85.4)	1.93 (1.78–2.10)	<0.001*	1.81 (1.62–2.01)	<0.001*
Current PPI user	10,231/37,904 (27.0)	27,673/37,904 (73.0)	4.17 (3.85–4.52)	<0.001*	3.70 (3.33–4.11)	<0.001*
Rural (n = 95,615)						
Past PPI user	5324/36,850 (14.5)	31,526/36,850 (85.6)	1.88 (1.75–2.03)	<0.001*	1.72 (1.56–1.90)	<0.001*
Current PPI user	12,938/48,296 (26.8)	35,358/48,296 (73.2)	4.08 (3.80–4.39)	<0.001*	3.47 (3.15–3.82)	<0.001*
Underweight (n = 4,400)						
Past PPI user	192/1,732 (11.1)	1540/1,732 (88.9)	1.58 (1.13–2.22)	0.008*	1.37 (0.88–2.14)	0.164
Current PPI user	474/2053 (23.1)	1579/2053 (76.9)	3.80 (2.76–5.24)	<0.001*	3.19 (2.06–4.97)	<0.001*
Normal weight (n = 62,015)						
Past PPI user	3427/25,357 (13.5)	21,930/25,357 (86.5)	2.06 (1.88–2.27)	<0.001*	1.91 (1.69–2.16)	<0.001*
Current PPI user	7650/29,046 (26.3)	21,396/29,046 (73.7)	4.72 (4.31–5.17)	<0.001*	4.11 (3.64–4.63)	<0.001*
Overweight (n = 46,654)						
Past PPI user	2856/18,288 (15.6)	15,432/18,288 (84.4)	1.90 (1.71–2.11)	<0.001*	1.75 (1.53–2.01)	<0.001*
Current PPI user	6454/23,306 (27.7)	16,852/23,306 (72.3)	3.92 (3.55–4.34)	<0.001*	3.42 (2.99–3.91)	<0.001*
Obese (n = 59,136)						
Past PPI user	3223/21,392 (15.1)	18,169/21,392 (84.9)	1.77 (1.60–1.94)	<0.001*	1.64 (1.45–1.86)	<0.001*
Current PPI user	8591/31,795 (27.0)	23,204/31,795 (73.0)	3.69 (3.36–4.04)	<0.001*	3.24 (2.87–3.66)	<0.001*
Nonsmoker (n = 128,525)						
Past PPI user	7197/49,268 (14.6)	42,071/49,268 (85.4)	2.00 (1.87–2.15)	<0.001*	1.84 (1.68–2.01)	<0.001*
Current PPI user	18,250/66,736 (27.4)	48,486/66,736 (72.7)	4.41 (4.12–4.71)	<0.001*	3.78 (3.47–4.13)	<0.001*
Past or current smoker (n = 43,680)						
Past PPI user	2501/17,501 (14.3)	15,000/17,501 (85.7)	1.73 (1.58–1.91)	<0.001*	1.65 (1.46–1.87)	<0.001*

Current PPI user	4919/19,464 (25.3)	14,545/19,464 (74.7)	3.52 (3.21–3.85)	<0.001*	3.21 (2.84–3.63)	<0.001*
Alcohol consumption < 1 time a week (n = 117,315)						
Past PPI user	6447/43,508 (14.8)	37,061/43,508 (85.2)	2.01 (1.87–2.16)	<0.001*	1.85 (1.69–2.03)	<0.001*
Current PPI user	17,209/62,275 (27.6)	45,066/62,275 (72.4)	4.41 (4.12–4.73)	<0.001*	3.85 (3.51–4.22)	<0.001*
Alcohol consumption ≥ 1 time a week (n = 54,890)						
Past PPI user	3251/23,261 (14.0)	20,010/23,261 (86.0)	1.75 (1.60–1.91)	<0.001*	1.63 (1.46–1.83)	<0.001*
Current PPI user	5960/23,925 (24.9)	17,965/23,925 (75.1)	3.56 (3.27–3.88)	<0.001*	3.16 (2.82–3.55)	<0.001*
Total cholesterol < 200 mg/dL (n = 89,569)						
Past PPI user	5149/34,879 (14.8)	29,730/34,879 (85.2)	1.90 (1.76–2.05)	<0.001*	1.76 (1.59–1.94)	<0.001*
Current PPI user	11,999/44,875 (26.7)	32,876/44,875 (73.3)	4.00 (3.72–4.31)	<0.001*	3.49 (3.17–3.86)	<0.001*
Total cholesterol ≥ 200 & < 240 mg/dL (n = 57,420)						
Past PPI user	3302/22,529 (14.7)	19,227/22,529 (85.3)	1.91 (1.74–2.10)	<0.001*	1.77 (1.56–2.00)	<0.001*
Current PPI user	7754/28,273 (27.4)	20,519/28,273 (72.6)	4.20 (3.83–4.60)	<0.001*	3.64 (3.22–4.11)	<0.001*
Total cholesterol ≥ 240 mg/dL (n = 25,216)						
Past PPI user	1247/9361 (13.3)	8114/9361 (86.7)	1.92 (1.64–2.23)	<0.001*	1.74 (1.43–2.12)	<0.001*
Current PPI user	3416/13,052 (26.2)	9636/13,052 (73.8)	4.42 (3.82–5.12)	<0.001*	3.73 (3.08–4.52)	<0.001*
SBP < 140 mmHg and DBP < 90 mmHg (n = 129,223)						
Past PPI user	7668/52,447 (14.6)	44,779/52,447 (85.4)	1.82 (1.71–1.94)	<0.001*	1.69 (1.56–1.83)	<0.001*
Current PPI user	17,280/61,769 (28.0)	44,489/61,769 (72.0)	4.13 (3.89–4.39)	<0.001*	3.55 (3.27–3.84)	<0.001*
SBP ≥ 140 mmHg or DBP ≥ 90 mmHg (n = 42,982)						
Past PPI user	2030/14,322 (14.2)	12,292/14,322 (85.8)	2.29 (2.01–2.60)	<0.001*	2.09 (1.77–2.46)	<0.001*
Current PPI user	5889/24,431 (24.1)	18,542/24,431 (75.9)	4.40 (3.88–4.97)	<0.001*	3.81 (3.25–4.47)	<0.001*

Fasting blood glucose < 100 mg/dL (n = 107,164)						
Past PPI user	6291/43,634 (14.4)	37,343/43,634 (85.6)	1.99 (1.85–2.13)	<0.001*	1.84 (1.67–2.01)	<0.001*
Current PPI user	14,396/51,511 (28.0)	37,115/51,511 (72.1)	4.57 (4.27–4.90)	<0.001*	3.91 (3.57–4.28)	<0.001*
Fasting blood glucose ≥ 100 mg/dL (n = 65,041)						
Past PPI user	3407/23,135 (14.7)	19,728/23,135 (85.3)	1.79 (1.64–1.96)	<0.001*	1.66 (1.48–1.87)	<0.001*
Current PPI user	8773/34,689 (25.3)	25,916/34,689 (74.7)	3.51 (3.22–3.82)	<0.001*	3.11 (2.78–3.49)	<0.001*

Abbreviations: BPPV, benign paroxysmal positional vertigo; CCI, Charlson comorbidity index; DBP, diastolic blood pressure; GERD, gastro-esophageal reflux disease; OW, overlap weighting; PPI, proton pump inhibitor; SBP, systolic blood pressure.

* Logistic regression model, Significance at $P < 0.05$.

† Adjusted for age, sex, income, region of residence, obesity, smoking status, alcohol consumption, total cholesterol, SBP, DBP, fasting blood glucose, CCI score, osteoporosis, prescription dates of H2 blocker, and the number of GERD treatment.

Table S2. Subgroup analyses regarding odds ratio (95% confidence intervals) of PPI prescription dates for BPPV according to age, sex, income, region of residence, obesity, smoking state, alcohol consumption, total cholesterol, blood pressure, and fasting blood glucose

**PPI prescription dates**	**BPPV**	**Comparison**	**Odds ratios (95% Confidence Intervals)**			
	**(Exposure/Total, %)**	**(Exposure/Total, %)**	**Crude**	***p*-Value**	**Adjusted Model with OW †**	***p*-Value**
Age < 60 years old (n = 72,790)						
≥1 days & <30 days	3736/25,004 (14.9)	21,268/25,004 (85.1)	2.30 (2.11–2.52)	<0.001*	2.21 (1.97–2.47)	<0.001*
≥30 days & < 365 days	7414/29,558 (25.1)	22,144/29,558 (74.9)	4.39 (4.03–4.78)	<0.001*	3.82 (3.42–4.26)	<0.001*
≥ 365 days	2787/9470 (29.4)	6683/9470 (70.6)	5.46 (4.98–6.00)	<0.001*	4.35 (3.83–4.94)	<0.001*
Age ≥ 60 years old (n = 99,415)						
≥1 days & <30 days	3680/22,551 (16.3)	18,871/22,551 (83.7)	1.95 (1.81–2.10)	<0.001*	1.82 (1.65–2.01)	<0.001*
≥30 days & < 365 days	7913/38,411 (20.6)	30,498/38,411 (79.4)	2.59 (2.42–2.78)	<0.001*	2.27 (2.07–2.50)	<0.001*
≥ 365 days	7337/27,975 (26.2)	20,638/27,975 (73.8)	3.55 (3.31–3.82)	<0.001*	2.87 (2.60–3.17)	<0.001*
Males (n = 73,470)						

≥1 days & <30 days	3591/20,840 (17.2)	17,249/20,840 (82.8)	2.13 (1.98–2.30)	<0.001*	2.02 (1.83–2.24)	<0.001*
≥30 days & < 365 days	5726/25,725 (22.3)	19,999/25,725 (77.7)	2.93 (2.73–3.16)	<0.001*	2.68 (2.43–2.95)	<0.001*
≥ 365 days	4448/16,455 (27.0)	12,007/16,455 (73.0)	3.80 (3.52–4.09)	<0.001*	3.26 (2.93–3.62)	<0.001*
Females (n = 98,735)						
≥1 days & <30 days	3825/26,715 (14.3)	22,890/26,715 (85.7)	2.11 (1.93–2.30)	<0.001*	1.94 (1.73–2.17)	<0.001*
≥30 days & < 365 days	9601/42,244 (22.7)	32,643/42,244 (77.3)	3.71 (3.42–4.04)	<0.001*	3.13 (2.81–3.49)	<0.001*
≥ 365 days	5676/20,990 (27.0)	15,314/20,990 (73.0)	4.68 (4.29–5.10)	<0.001*	3.72 (3.32–4.18)	<0.001*
Low income (n = 77,790)						
≥1 days & <30 days	3236/21,274 (15.2)	18,038/21,274 (84.8)	2.14 (1.97–2.33)	<0.001*	2.01 (1.80–2.24)	<0.001*
≥30 days & < 365 days	7109/31,066 (22.9)	23,957/31,066 (77.1)	3.54 (3.27–3.85)	<0.001*	3.09 (2.78–3.44)	<0.001*
≥ 365 days	4514/16,398 (27.5)	11,884/16,398 (72.5)	4.54 (4.17–4.94)	<0.001*	3.67 (3.27–4.12)	<0.001*
High income (n = 94,415)						
≥1 days & <30 days	4180/26,281 (15.9)	22,101/26,281 (84.1)	2.01 (1.86–2.17)	<0.001*	1.90 (1.72–2.10)	<0.001*
≥30 days & < 365 days	8218/36,903 (22.3)	28,685/36,903 (77.7)	3.05 (2.83–3.28)	<0.001*	2.72 (2.47–3.00)	<0.001*
≥ 365 days	5610/21,047 (26.7)	15,437/21,047 (73.4)	3.87 (3.58–4.17)	<0.001*	3.28 (2.96–3.64)	<0.001*
Urban (n = 76,590)						
≥1 days & <30 days	3400/21,351 (15.9)	17,951/21,351 (84.1)	2.14 (1.97–2.33)	<0.001*	2.04 (1.83–2.27)	<0.001*
≥30 days & < 365 days	6578/29,230 (22.5)	22,652/29,230 (77.5)	3.28 (3.02–3.56)	<0.001*	2.94 (2.64–3.27)	<0.001*
≥ 365 days	4627/17,242 (26.8)	12,615/17,242 (73.2)	4.14 (3.81–4.50)	<0.001*	3.56 (3.17–3.99)	<0.001*
Rural (n = 95,615)						
≥1 days & <30 days	4016/26,204 (15.3)	22,188/26,204 (84.7)	2.02 (1.87–2.18)	<0.001*	1.88 (1.70–2.08)	<0.001*
≥30 days & < 365 days	8749/38,739 (22.6)	29,990/38,739 (77.4)	3.25 (3.02–3.50)	<0.001*	2.83 (2.57–3.12)	<0.001*

≥ 365 days	5497/20,203 (27.2)	14,706/20,203 (72.8)	4.17 (3.86–4.50)	<0.001*	3.37 (3.03–3.74)	<0.001*
Underweight (n = 4400)						
≥1 days & <30 days	162/1296 (12.5)	1134/1296 (87.5)	1.81 (1.28–2.56)	0.001*	1.63 (1.03–2.56)	0.036*
≥30 days & < 365 days	328/1761 (18.6)	1433/1761 (81.4)	2.90 (2.09–4.02)	<0.001*	2.46 (1.58–3.83)	<0.001*
≥ 365 days	176/728 (24.2)	552/728 (75.8)	4.04 (2.85–5.72)	<0.001*	3.04 (1.83–5.06)	<0.001*
Normal weight (n = 62,015)						
≥1 days & <30 days	2716/19,004 (14.3)	16,288/19,004 (85.7)	2.20 (2.00–2.43)	<0.001*	2.09 (1.84–2.36)	<0.001*
≥30 days & < 365 days	5458/24,470 (22.3)	19,012/24,470 (77.7)	3.79 (3.45–4.16)	<0.001*	3.35 (2.97–3.78)	<0.001*
≥ 365 days	2903/10,929 (26.6)	8026/10,929 (73.4)	4.78 (4.33–5.26)	<0.001*	3.98 (3.48–4.55)	<0.001*
Overweight (n = 46,654)						
≥1 days & <30 days	2174/12,991 (16.7)	10,817/12,991 (83.3)	2.06 (1.85–2.29)	<0.001*	1.93 (1.68–2.22)	<0.001*
≥30 days & < 365 days	4323/18,457 (23.4)	14,134/18,457 (76.6)	3.13 (2.83–3.47)	<0.001*	2.78 (2.42–3.18)	<0.001*
≥ 365 days	2813/10,146 (27.7)	7333/10,146 (72.3)	3.93 (3.53–4.37)	<0.001*	3.31 (2.86–3.83)	<0.001*
Obese (n = 59,136)						
≥1 days & <30 days	2364/14,264 (16.6)	11,900/14,264 (83.4)	1.98 (1.79–2.18)	<0.001*	1.86 (1.64–2.12)	<0.001*
≥30 days & < 365 days	5218/23,281 (22.4)	18,063/23,281 (77.6)	2.88 (2.62–3.16)	<0.001*	2.58 (2.28–2.91)	<0.001*
≥ 365 days	4232/15,642 (27.1)	11,410/15,642 (72.9)	3.69 (3.36–4.06)	<0.001*	3.13 (2.75–3.56)	<0.001*
Nonsmoker (n = 128,525)						
≥1 days & <30 days	5400/34,825 (15.5)	29,425/34,825 (84.5)	2.15 (2.00–2.31)	<0.001*	2.02 (1.84–2.21)	<0.001*
≥30 days & < 365 days	12,210/52,729 (23.2)	40,519/52,729 (76.8)	3.53 (3.30–3.78)	<0.001*	3.09 (2.83–3.38)	<0.001*
≥ 365 days	7837/28,450 (27.6)	20,613/28,450 (72.5)	4.45 (4.15–4.77)	<0.001*	3.68 (3.35–4.04)	<0.001*
Past or current smoker (n = 43,680)						

≥1 days & <30 days	2016/12,730 (15.8)	10,714/12,730 (84.2)	1.96 (1.78–2.16)	<0.001*	1.88 (1.66–2.14)	<0.001*
≥30 days & < 365 days	3117/15,240 (20.5)	12,123/15,240 (79.6)	2.67 (2.44–2.94)	<0.001*	2.50 (2.21–2.83)	<0.001*
≥ 365 days	2287/8995 (25.4)	6708/8995 (74.6)	3.55 (3.22–3.91)	<0.001*	3.07 (2.68–3.53)	<0.001*
Alcohol consumption < 1 time a week (n = 117,315)						
≥1 days & <30 days	4940/31,328 (15.8)	26,388/31,328 (84.2)	2.16 (2.01–2.33)	<0.001*	2.04 (1.86–2.24)	<0.001*
≥30 days & < 365 days	11,303/47,840 (23.6)	36,537/47,840 (76.4)	3.57 (3.33–3.84)	<0.001*	3.18 (2.90–3.49)	<0.001*
≥ 365 days	7413/26,615 (27.9)	19,202/26,615 (72.2)	4.46 (4.15–4.80)	<0.001*	3.75 (3.40–4.14)	<0.001*
Alcohol consumption ≥ 1 time a week (n = 54,890)						
≥1 days & <30 days	2476/16,227 (15.3)	13,751/16,227 (84.7)	1.93 (1.77–2.12)	<0.001*	1.85 (1.65–2.09)	<0.001*
≥30 days & < 365 days	4024/20,129 (20.0)	16,105/20,129 (80.0)	2.68 (2.46–2.93)	<0.001*	2.43 (2.17–2.73)	<0.001*
≥ 365 days	2711/10,830 (25.0)	8119/10,830 (75.0)	3.59 (3.28–3.93)	<0.001*	3.03 (2.67–3.45)	<0.001*
Total cholesterol < 200 mg/dL (n = 89,569)						
≥1 days & <30 days	4030/24,883 (16.2)	20,853/24,883 (83.8)	2.12 (1.96–2.29)	<0.001*	1.99 (1.80–2.21)	<0.001*
≥30 days & < 365 days	7596/34,056 (22.3)	26,460/34,056 (77.7)	3.15 (2.92–3.40)	<0.001*	2.79 (2.53–3.09)	<0.001*
≥ 365 days	5522/20,815 (26.5)	15,293/20,815 (73.5)	3.96 (3.66–4.28)	<0.001*	3.31 (2.98–3.69)	<0.001*
Total cholesterol ≥ 200 & < 240 mg/dL (n = 57,420)						
≥1 days & <30 days	2476/16,309 (15.2)	13,833/16,309 (84.8)	1.99 (1.81–2.19)	<0.001*	1.89 (1.66–2.14)	<0.001*
≥30 days & < 365 days	5424/23,159 (23.4)	17,735/23,159 (76.6)	3.40 (3.10–3.73)	<0.001*	3.01 (2.66–3.40)	<0.001*
≥ 365 days	3156/11,334 (27.9)	8178/11,334 (72.2)	4.29 (3.89–4.73)	<0.001*	3.58 (3.13–4.09)	<0.001*
Total cholesterol ≥ 240 mg/dL (n = 25,216)						
≥1 days & <30 days	910/6363 (14.3)	5453/6363 (85.7)	2.08 (1.78–2.44)	<0.001*	1.95 (1.59–2.39)	<0.001*
≥30 days & < 365 days	2307/10,754 (21.5)	8447/10,754 (78.6)	3.41 (2.94–3.95)	<0.001*	2.95 (2.43–3.57)	<0.001*

≥ 365 days	1446/5296 (27.3)	3850/5296 (72.7)	4.68 (4.02–5.46)	<0.001*	3.79 (3.07–4.68)	<0.001*
SBP < 140 mmHg and DBP < 90 mmHg (n = 129,223)						
≥1 days & <30 days	6124/38,634 (15.9)	32,510/38,634 (84.2)	2.00 (1.88–2.14)	<0.001*	1.90 (1.75–2.07)	<0.001*
≥30 days & < 365 days	11,792/50,293 (23.5)	38,501/50,293 (76.6)	3.26 (3.07–3.46)	<0.001*	2.85 (2.63–3.09)	<0.001*
≥ 365 days	7032/25,289 (27.8)	18,257/25,289 (72.2)	4.10 (3.85–4.37)	<0.001*	3.31 (3.03–3.62)	<0.001*
SBP ≥ 140 mmHg or DBP ≥ 90 mmHg (n = 42,982)						
≥1 days & <30 days	1292/8921 (14.5)	7629/8921 (85.5)	2.34 (2.05–2.68)	<0.001*	2.18 (1.84–2.59)	<0.001*
≥30 days & < 365 days	3535/17,676 (20.0)	14,141/17,676 (80.0)	3.46 (3.05–3.92)	<0.001*	3.12 (2.66–3.67)	<0.001*
≥ 365 days	3092/12,156 (25.4)	9064/12,156 (74.6)	4.72 (4.16–5.36)	<0.001*	3.97 (3.36–4.69)	<0.001*
Fasting blood glucose < 100 mg/dL (n = 107,164)						
≥1 days & <30 days	4828/32,006 (15.1)	27,178/32,006 (84.9)	2.10 (1.95–2.25)	<0.001*	1.99 (1.81–2.19)	<0.001*
≥30 days & < 365 days	10,206/43,362 (23.5)	33,156/43,362 (76.5)	3.63 (3.38–3.89)	<0.001*	3.19 (2.91–3.49)	<0.001*
≥ 365 days	5653/19,777 (28.6)	14,124/19,777 (71.4)	4.72 (4.39–5.08)	<0.001*	3.82 (3.45–4.22)	<0.001*
Fasting blood glucose ≥ 100 mg/dL (n = 65,041)						
≥1 days & <30 days	2588/15,549 (16.6)	12,961/15,549 (83.4)	2.07 (1.89–2.27)	<0.001*	1.94 (1.72–2.19)	<0.001*
≥30 days & < 365 days	5121/24,607 (20.8)	19,486/24,607 (79.2)	2.72 (2.50–2.97)	<0.001*	2.45 (2.19–2.76)	<0.001*
≥ 365 days	4471/17,668 (25.3)	13,197/17,668 (74.7)	3.51 (3.21–3.84)	<0.001*	2.98 (2.64–3.36)	<0.001*

Abbreviations: BPPV, benign paroxysmal positional vertigo; CCI, Charlson comorbidity index; DBP, diastolic blood pressure; GERD, gastro-esophageal reflux disease; OW, overlap weighting; PPI, proton pump inhibitor; SBP, systolic blood pressure.

* Logistic regression model, Significance at $P < 0.05$.

† Adjusted for age, sex, income, region of residence, obesity, smoking status, alcohol consumption, total cholesterol, SBP, DBP, fasting blood glucose, CCI score, osteoporosis, prescription dates of H2 blocker, and the number of GERD treatment.
